# Current state of ethical challenges reported in Saudi Arabia: a systematic review & bibliometric analysis from 2010 to 2021

**DOI:** 10.1186/s12910-022-00816-6

**Published:** 2022-08-13

**Authors:** Alexander Woodman, Khawaja Bilal Waheed, Mohammad Rasheed, Shakil Ahmad

**Affiliations:** 1 Yale Interdisciplinary Center for Bioethics, Yale University, New Haven, CT USA; 2 Radiodiagnostic and Imaging Department, King Fahad Military Medical Complex, Dhahran, Saudi Arabia; 3 Vice Deanship of Postgraduate Studies and Research, Prince Sultan Military College of Health Sciences, Dhahran, Saudi Arabia; 4 Deanship of Library Affairs, Imam Abdulrahman Bin Faisal University, Dammam, Saudi Arabia

**Keywords:** Ethical challenges, Medical ethics curriculum, Doctor-patient relationship, Informed consent, Do-not-resuscitate, Organ donation and transplantation, Systematic review, Bibliometric analysis, Saudi Arabia

## Abstract

**Background:**

Over the past few years, five domains of importance about the current state of bioethics in Saudi Arabia have shaped the perspective of most research: doctor-patient relationship, informed consent, do-not-resuscitate, organ donation, and transplantation, medical students’ knowledge and attitudes about medical ethics curriculum. This systematic review aimed to systematically identify, compile, describe and discuss ethical arguments and concepts in the best-studied domains of bioethics in Saudi Arabia and to present cultural, social, educational, and humane perspectives.

**Methods:**

Six databases were searched using Boolean operators (PubMed, Embase, Web of Science, Scopus, CINAHL, Google Scholar) from December 2020–June 2021. The search and report process followed the statement and flowchart of preferred reporting items for systematic reviews and meta-analyses (PRISMA).

**Resutls:**

The search resulted in 1651 articles, of which 82 studies were selected for a final review and assessment. There is a gradual increase in research, whereby a substantial increase was observed from 2017. Most of the published articles focused on ‘Organ Donation & Transplantation’ with 33 articles, followed by ‘Doctor-Patient Relations’ with 18 publications. Most of the published articles were from Central Province (33), followed by Western Province (16). The authorship pattern showed a collaborative approach among researchers. The thematic analysis of keywords analysis showed that ‘Saudi Arabia,’ ‘attitude PHC,’ ‘organ donation,’ ‘knowledge and education,’ and ‘donation’ have been used the most commonly.

**Conclusion:**

This systematic quantitative synthesis is expected to guide researchers, stakeholders, and policymakers about the strengths and gaps in knowledge and attitudes regarding medical ethics in Saudi Arabia, both among the general public and health professionals.

## Background

The purpose of biomedical ethics is to study, build, and judge the criteria necessary to philosophically assess medical quandaries that demand otherwise morally gray or seemingly immoral solutions [1, 2]. Beauchamp and Childress (1979) proposed developing moral judgment in bioethics in terms of four principles (prima facie) that are a part of common morality (unphilosophical commonsense and tradition) and are neither overarching nor overly specific: autonomy, beneficence, non-maleficence, and justice [2]. These principles are also a part of the Islamic bioethics present in Saudi Arabia [2, 4]. Thus, Islamic bioethics or Islamic medical ethics is a system of moral assessment made to identify, analyze, and solve ethical problems arising in medical practice and research based on Islamic moral and legislative sources (primarily Quran and Sunna and secondarily by ijma/ consensus of Muslim scholars, qiyas/ analogies from Quran or legal precedents) [2, 4].

There is extensive literature on theoretical discussions and fundamental issues to understand the current debates on bioethics within the Islamic moral and legislative sources as well as within Saudi healthcare system [2–4] Hence a preliminary search of databases was conducted to look into most studied areas of bioethics in Saudi Arabia. The search showed that there are a few fundamental domains of importance to understanding the current state of bioethics in Saudi Arabia that have shaped the perspective of most research [3–5].

The first concern is physician–patient relations, which sets clear boundaries on what doctors are allowed and not allowed to do. According to Saudi Commission for Health Specialties (2015), the core of every successful physician–patient interaction is trust, integrity, and honesty. Healthcare professionals must be upfront with patients regarding their rights and responsibilities, and how those rights are supported through informed consent [4–6]. However, the physician–patient relationship in Saudi Arabia is different as Saudi patients cannot be isolated from their families. The family plays an essential role in decision-making in Saudi Arabia, and doctors recognize the importance of this domain in bioethics and its relation to the patient’s family members [6–9]. Similar to decision-making, Saudi doctors recognize the importance of informed consent and its relation to the patient’s family members. In this context, informed consent aims to inform not only the patient but the family of the expected consequences of the treatment course [8–10].

Illness in Islam is considered a trial through which the patient must persevere, and the doctor has no right to terminate a patient's life. When death becomes inevitable, as determined by the doctors assigned to terminally ill patients, the *Fatwa*^1^ No. 12086 dated 30/6/1409 (1989) of the High Council of Scholars and *Ifta'a*^2^ of Saudi Arabia, Riyadh, allowed the "Do Not Resuscitate Policy" if three competent doctors deemed the available medical interventions to be futile (Permanent Committee for Scholarly Research and Ifta’a, 1989). The decisions and integrity of these specialists and competent doctors are to be respected [10]. The family must be informed of the decision, but they cannot interfere, as they are not considered qualified under the *Fatwa*. The *Fatwa* must be explained to the family. If the family still insists against the DNR policies, they should be offered to transfer the patient to a different hospital willing to accept the patient. Despite existing regulations and legislation, the debate about DNR practice continues to vary widely [6, 10].

The fourth most debated bioethical domain is organ transplantation, a common practice since the 1960s. Organ donation in Saudi Arabia has been rising, as awareness of this global phenomenon, which aims to save lives through altruism, has increased [6]. In 1985, the Saudi Center of Organ Transplantation (SCOT) was founded as the government agency. At present, SCOT oversees all national organ donation and transplantation activities in Saudi Arabia [11, 12]. Nevertheless, organ transplantation raises several ethical issues among healthcare providers and general public, including the definition of death, the decision to donate organs, organ procurement, and organ allocation [6, 13, 14].

In general, the development of medical ethics and its domains have been accompanied by numerous social, legal, and ethical debates [1–4]. Public opinion tends to be skeptical about such developments in science and is prone to showing significant ambivalence in their attitude. Moreover, the variance in ethical education in Saudi Arabia and other Middle Eastern countries reflects knowledge gaps among both undergraduate and postgraduate medical students and, in some cases, doctors [1, 2]. Bioethics curriculum development was seen as part of general steps forward in developing moral attitudes, the complexity of the healthcare system, ethical and legal issues that need to be addressed systematically and consistently through the integration into medical education [2–5]. This shift has resulted in the increasing involvement of scientists and clinicians in teaching medical ethics based on the ideas of the philosophers who pioneered the teaching of the subject [2–5].

While modern research in bioethics covers issues such as stem cell research, genetic and biobanking, cloning, and fertility treatment, the search for evidence showed that despite well-establsihed *fatwas*, bioethics in Saudi Arabia is still on the rise, exploring the very foundations of medical ethics. Hence, this research cannot claim to have treated all domains of bioethics within the Saudi healthcare system. Rather, the aim is to present fundamental domains of bioethics that will shed light on essential ethical questions and applied ethics.These fundamental bioethical domains often challenge researchers and general public, as they contradict moral traditions and sometimes perceive matters in an unconventional light, which must take the entire spectrum of ethical theories into account [1, 2]. For this reason, this systematic review aimed to systematically identify, compile, describe and discuss ethical arguments and concepts in the most-argued domains of bioethics in Saudi Arabia and present cultural, social, educational, and humane perspectives.

## Methods

Systematic reviews are best known as a consolidation of sources that help researchers to identify what is known with certainty, what is tentatively known, and where the gaps in knowledge are. The goal of systematic review is to reach broad conclusions that represent the findings of individual studies as a body of work, which is often referred to as synthesis [15–17]. In order to meet the goals of contemporary systematic reviews, a straightforward, transparent, and reproducible approach has been adapted, recommended, and used by previous researchers. This approach is essential for generating reliable data for future research and prospective policy development [18–20].

This systematic review has been carried in accordance with the following stages: (1) creating clear and explicit objectives, (2) definition of inclusion and exclusion criteria based on the objectives of the study and the selected the domains, (3) performing an effective and wide-ranging literature search based on the objectives and eligibility criteria, (4) exercising a methodological quality of studies, (5) extracting data from included studies, and (6) presenting findings of the review in the form of a descriptive summary in a table supplemented with text commentary [18–20].

## Research aims and questions

To the authors’ knowledge, there are no previous studies aimed to systematically identify, compile, describe and discuss ethical arguments and concepts in the best-studied domains of bioethics in Saudi Arabia and to present cultural, social, educational, and humane perspectives. Based on the aim of the study, the following questions were developed:

1. What do Saudi health professionals and the general public know about *fatwas* regulating the medical ethics curriculum, doctor-patient relationship, informed consent, DNR, and organ transplantation?
2. What is the attitude of Saudi health professionals and the general public about medical ethics curriculum, doctor-patient relationship, informed consent, DNR, and organ transplantation?

What are the concerns of Saudi health professionals and students about medical ethics curriculum, doctor-patient relationship, informed consent, DNR, and organ transplantation?

### Eligibility criteria

It is essential to specify the eligibility criteria (i.e., inclusion/exclusion) for studies that need to be met before being included in a systematic review. Failure to identify eligibility criteria can result in bias. The eligibility criteria should derive from the aims and research questions [12, 17, 21].

The inclusion and exclusion criteria of the current systematic review are as follows:

#### Inclusion criteria

- Publication Type: Published journal articles
- Language: Title/abstract level: only articles with at least an abstract in English
- Be explicitly concerned with normative ethical considerations of medical topics in Saudi Arabia
  i. Pose an ethical question
  ii. Determine ethical problems/challenges
  iii. Address ethical decision making or the use of ethical frameworks for decision making
  iv. Explore ethical views or reasons for/against a decision
  v. Look for/produce experimental data for ethical decision making or ethical evaluation
  vi. Examine ethical regulations or recommendations
  vii. Studies highlighting or emphasizing upon physician–patient relationship (specifically informed consent), beneficence (DNR, organ donation and transplantation), non-malificence (physician competency, standard of care) and justice (provision of care) were selected.

#### Exclusion criteria

- Articles published before 2010
- Randomized controlled trials (RCTs)
- Cohort studies
- Case–control studies
- Reviews and editorials
- Meta-analyses

The publication is written in a language other than English, e.g., Arabic.

### Literature search

Six electronic literature databases were searched, covering the fields of healthcare sciences, ethics, and religion: PubMed, Embase, Web of Science, Scopus, CINAHL, Google Scholar. All databases were searched using Boolean operators (AND, OR, NOT) expressed in English through a combination of words in a single search. For instance, Ethical issues truth AND Saudi Arabia Ethical issues AND euthanasia OR do not resuscitate AND Saudi Arabia Islam ethics and organ donation OR transplantation and Saudi Arabia Medical ethics AND privacy confidentiality AND Saudi Arabia DNR OR DNT AND Saudi Arabia Do not resuscitate OR do not treat AND Saudi Arabia Medical issues OR bioethics AND Saudi Arabia. In addition to applying Boolean operators, National Center for Biotechnology Information (NCBI) filters were applied. This allowed to search for articles by type, year(s) of interest, species (i.e., humans), age, language, and other filters. The two search strings used for the PubMed database are presented in Table 1. An independent librarian at Prince Sultan Military College of Health Sciences (PSMCHS) reviewed and validated the final search strategies.

**Table 1:** PubMed search strings stratified according to organizing concepts—explorative and refined

*PubMed explorative search*	
Date	December 2020–June 2021
Publication dates	2010–2021
Language	English
Search string	"Saudi Arabia"[Mesh]) AND "Bioethics"[Mesh]) AND "Ethics, Medical"[Mesh]) AND "Ethics, Professional"[Mesh]) OR "Ethics, Professional/legislation and jurisprudence"[Mesh]
*PubMed explorative search*	
Date	December 2020–June 2021
Publication dates	2010–2021
Language	English
	"Saudi Arabia"[Mesh]) AND "Informed Consent"[Mesh]) AND "Consent Forms"[Mesh]) AND "Doctor-Patient Relations/ethics"[Mesh] OR "Doctor-Patient Relations/standards"[Mesh] AND "Delivery of Health Care/ethics"[Mesh] AND "Advance Directive Adherence"[Mesh] AND "Resuscitation Orders"[Mesh] AND "Tissue Donors/education" [Mesh] OR "Tissue Donors/ethics"[Mesh] AND "Organ Transplantation"[Mesh]) AND "Ethics, Professional"[Mesh] OR "Ethics Committees"[Mesh] OR "Ethics, Clinical"[Mesh]

### Selection process

**Fig. 1:**
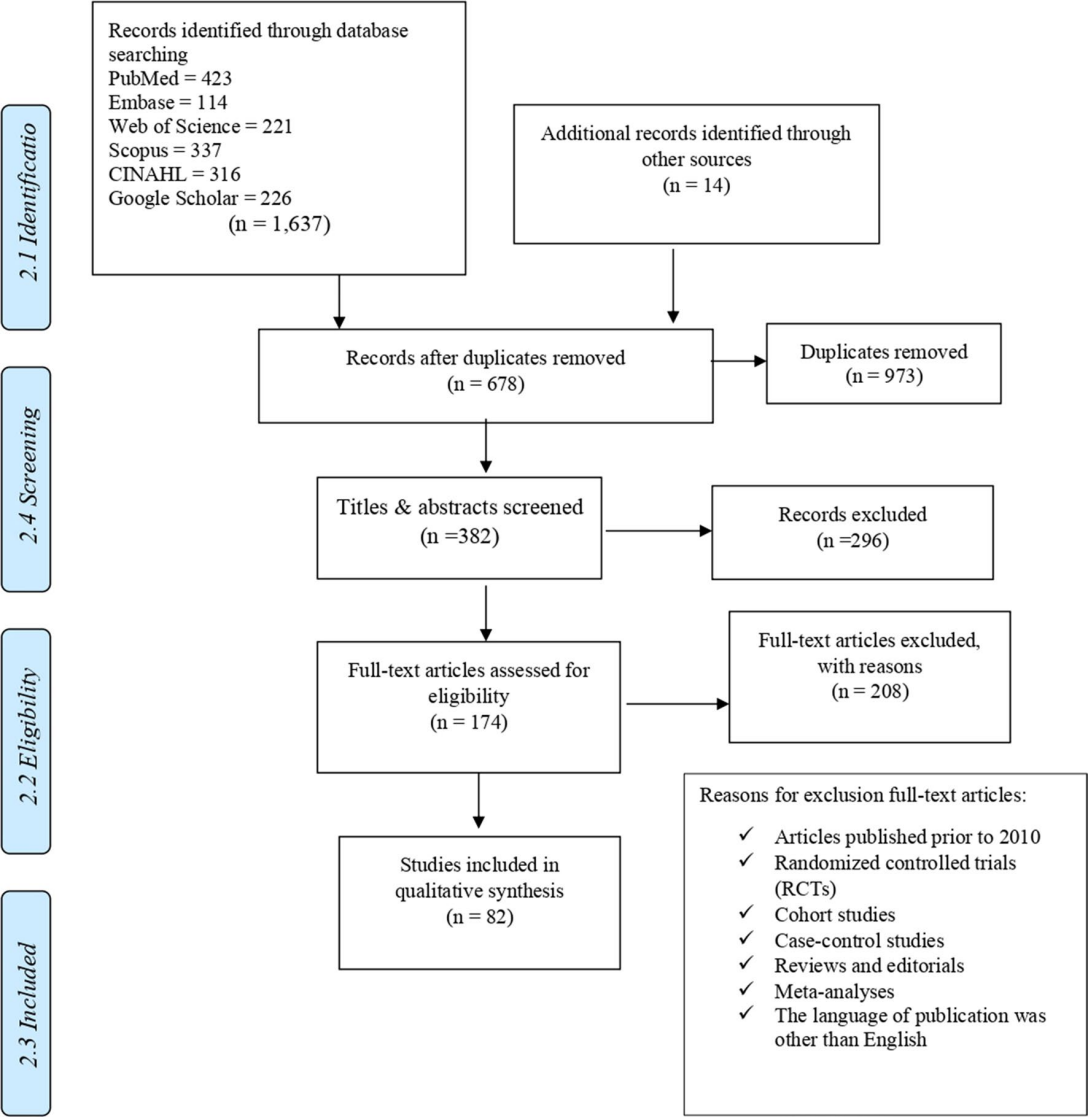
Study identification, screening, and inclusion, guided by PRISMA

The database was searched between December 2020 and June 2021. The first author originally made the selection. The second author then checked all selection results for inclusion and exclusion criteria. To reach a consensus, discrepancies were discussed and successfully resolved through discussion with the third author. The search and report process followed the statement and flowchart of Preferred Reporting Items for Systematic Reviews and Meta-Analyses (PRISMA) (Fig. 1). The full texts of potentially eligible records were retrieved and independently assessed for eligibility by the first and second authors.

## Results

The database search resulted in 1,637 studies. Fourteen articles were included, sourced from the reference lists identified through the original database search. After removing duplicates (973), the relevant articles and publications were selected in two stages. During the first stage, the titles and abstracts of the articles were screened, and non-relevant articles were excluded (296). In the second stage, the full text of included studies was explored. Articles published before 2010, randomized controlled trials, cohort studies, case–control studies, reviews and editorials, meta-analyses, and publication language was other than English were excluded (208). As a result, 82 studies were selected for a final review and assessment (Fig. 1).

### Data extraction

The following data items were extracted from the included studies and reported in Table 2: (1) author and year of publication of the study; (2) study setting, sample size, and characteristics of participants; (3) main findings of the research. An additional column has been added to show the definition and rules of the given bioethics domain in the Saudi setting (Table 2).

**Table 2:** Synthesis of included studies following domains

Definition of domain	Author	Study setting	Findings
*Medical ethics curriculum in Saudi Arabia*			
Ethics is an emerging discipline in many medical schools in the Islamic world, being taught in some schools by non-specialists with limited experience in teaching ethics and not housed in proper ethics departments. Teaching medical ethics at the undergraduate level will expand the knowledge of the standards of professional conduct and prepare the graduates to face the ethical dilemmas arising from progressive advances in medical practice and science applications. In the Islamic world, medical school curricula should contain a study of the Islamic code of medical ethics. Islamic law is a compendium of ethics, morality, and legal rules. Islam considers medical ethics the same as ethics in other aspects of life. The syllabus should be logical and practical, parallel to the basic medical study program (Chamsi-Pasha & Al-Bar, 2016)	Al-Haqwi, and Al-Shehri [22]	Medical students (n = 41) from Riyadh	Strong agreement on the importance of learning the principles of medical ethics
			To a lesser extent, the contents of the course were relevant to Saudi culture
			A disagreement with the view that medical ethics was just common sense
			Participants could not decide whether the assessment methods were appropriate or not
	Darwish and Sabra [23]	Medical students (n = 164) from Dammam	None of the students had received ethics courses in their basic study
			Only a few attended training courses about medical ethics
			The majority recognized that disclosure of patient information by a doctor is allowed if it would be beneficial to the society
			The majority wrongly mentioned that patient information can be disclosed to a third party without the patient's consent
	Aldughaither et al. [24]	Medical students (n = 250) from Riyadh	The majority were satisfied with the course and timing of medical ethics
			The method of instruction should be changed to case-based teaching
			Ethical issues based on Islamic *Fiqh* should continue to be discussed
			The majority wished to discuss issues related to the doctor-patient relationship and professionalism
			Preferred topics were brain death, organ transplantation, cosmetic surgery, abortion, terminal care, reproduction, doctors’ rights, end-of-life issues, and medical errors
	Al Kabba et al. [25]	14 public medical schools in Saudi Arabia	All schools had a curriculum dedicated to medical ethics
			Six schools had no ethics departments; three had a separate ethics department, five taught ethics as part of another department—family or community medicine
			Thirteen schools made learning medical ethics compulsory for students; five schools had taught ethics as an independent course; four as part of another course
			Lack of guidance on how to develop a uniform curriculum that addresses both the religious aspects and the practical professional aspects that are sourced from western or other non-Islamic sources to prepare the graduates to practice in non-Muslim countries, as well as dealing with non-Muslim patients
			Medical licensing bodies should establish benchmarks to guide the medical schools in the formulation of their curricula
	El-Sobkey et al. [26]	Health professions’ students (n = 239) from Riyadh	The majority believed in the ineffectiveness of patient’s rights
			Half of the sample had perceptual knowledge about the Saudi Patient’s Bill of Rights (PBR)
			Only a few participants knew some items of PBR
			Only one course within the curriculum was related to patient’s rights
	Alyousefi et al. [27]	Students (n = 247) from Riyadh	Participants could recognize the ethical issues indirectly reflecting moral sensitivity
			The majority were able to utilize the ethical principles they learned during the course and apply these principles for the analyses of the cases
			Participants were able to recognize, analyze, and criticize unethical and unprofessional behaviors
			Participants had less experience in issues such as end-of-life, brain death, assisted reproduction, DNR
	Ghamri and Al-Raddadi [28]	Residents (n = 88) from Jeddah	The majority agreed that ethics should be taught in the residency curriculum
			Less than half agreed that there were no right/wrong answers to ethical questions
			The majority agreed that ethics is a discipline with its methods, literature, vocabulary, and content
			The majority disagreed with the statement that training in ethics did not help residents deal with ethical conflicts
			Only a few of participants agreed that the training was adequate to handle the ethical conflicts they were likely to face
	AbuAbah et al. [29]	Doctors (n = 200) from Riyadh	The majority received only theoretical teaching on ethics during medical school
			Theoretical teaching does not address the practical dilemma after graduation
			The majority knew about the policy on DNR decision making, orders, and documentation
			A lack of knowledge about organ donation regulations, withholding or stopping mechanical ventilation, conflict with family, and advice from the ethics committee
			The majority required clear guidelines to make appropriate ethical decisions
	Alnajjar et al. [30]	Students (n = 113) from Jeddah	More than half of the participants understood the concept of brain death
			Uncertainty on whether brain death was synonymous with the patient demise
			The majority was aware of the importance of organ donation
	Alotaibi et al. [31]	Dental researchers (n = 660) from dental colleges of Saudi Arabia	A lack of knowledge in research ethics and need to attend courses/ workshops
			The importance of teaching research ethics as a mandatory course and training all specialists in research ethics
			Positive attitudes towards ethics committees to review human research projects
*Doctor-patient relations*			
At the core of any successful doctor-patient interaction is trust. Integrity and honesty are necessary to achieve the trust of patients. Patients do not expect perfection in their care providers but need to know that their doctor is reliable and trustworthy. As communicators, doctors effectively facilitate the doctor-patient relationship and the dynamic exchanges before, during, and after the medical encounter (Saudi Commission for Health Specialties, 2015)	Alghanim [32]	Patients, doctors, and nurses (n = 799) from Riyadh	More than three quarters of patients and one-third of doctors and nurses did not know about PBR
			Those who knew about PBR had little knowledge about the bill contents
			Health personnel dissatisfaction, insufficient number of staff, and lack of essential facilities in primary health care centers were obstacles in implementing PBR
	Al Qarny et al. [33]	Health care workers (n = 224) from Taif	Almost half of the participants had little knowledge regarding medical ethics in general
			Nurses reported more significant knowledge of medical ethics compared to doctors
			The majority knew that every patient must be treated with honesty and dignity
			Illegal pregnancy abortion cannot be recommended
			Patients with high socioeconomic class should not be treated with special care
			The majority knew that confidentiality should be kept and the patient should always be told if something was wrong
			Children should never be treated without the consent of their parents or guardians (except in an emergency)
			Almost two-thirds knew that doctors and nurses should not refuse to treat patients who behave violently
	Al-Yousefi [34]	Doctors (n = 225) from Riyadh	The majority reported the positive influence of religion on health and in coping with diseases
			Religion rarely/never causes guilt, anxiety, or other negative emotions that lead to an increased patient suffering
			Patients frequently used religion to avoid taking responsibility for their health
			Family doctors were more likely to initiate religious discussions
			Doctors with intrinsic religiosity were more likely to share their religious views
	Al-Mohaimeed and Sharaf [35]	Doctors (n = 458) from Qassim	Most doctors did not avoid telling their patients bad news
			Almost half of the hospital doctors and a third of PHC doctors believed that the patient has the right to know the diagnosis
			Doctors found it easier to speak to the relatives of patients than the patients
			Doctors with higher qualifications had lower total scores in breaking bad news
	Saleh and Khereldeen [36]	Doctors (n = 246) from Mecca	All participants indicated that the right to know the name of a doctor, to be treated with care and respect, the right to know treatment alternatives is among patient’s rights
			Most doctors were aware of patients’ rights to respect, privacy, and confidentiality
			A few participants reported that patients' rights were maintained
			A few participants agreed that patients have the right to access their medical records
	Fayez et al. [37]	Health care workers (n = 370) from four tertiary hospitals in Saudi Arabia	The majority agreed that unethical behavior occurred in their workplace
			Confidentiality was compromised, informed consent was not being handled properly, and bad news was not delivered with the care it requires
			Doctors lacking empathy, patient autonomy was not fully respected
			Discrimination at the workplace
			Senior staff does not give enough consideration to ethical issues related to patients
			The residents were more likely to agree that unethical behaviors occur than staff
	Al Shahrani et al. [38]	Patients (n = 72) from Abha	The highest satisfaction rank was with the patient’s expectations of treatment results followed by the work environment and reception and doctor-patient relationship
			Patients were delighted with the technical aspects of the treatment
			Most patients were dissatisfied with the explanation of the procedure during treatment
	Al-Zahrani et al. [39]	Primary health care doctors (n = 70) from Riyadh	The majority thought that lack of training, cultural norms, gender difference, and lack of time were the main barriers to applying effective communication with patients
			Using non-verbal cues to communicate with patients were used rarely
			There was no correlation between knowledge and practice of communication skills
			Doctors who consciously applied the communication skills in their practice scored better in daily practice
			Specialized and MBBS/MD doctors were more confident in their self-rating of communication skills
			The majority of residents evaluated their communication skills with lesser self-confidence
	Al Ali and Elzubair [40]	Attendees (n = 374) & Doctors (n = 27) from Dammam	Attendees’ satisfaction with doctors’ empathy was not high
			Elderly attendees and those with little education indicated greater satisfaction than younger and more educated attendees
			A specialty of the doctor-affected communication and patient satisfaction
			Family doctors were more closely linked to rapport building, psychosocial exchange, and patient-centeredness
			The high professional status of a doctor was positively related to satisfaction with doctors’ empathy
	Banaser et al. [41]	Adult patients (n = 22) from Riyadh	The majority were optimistic about their experiences in a doctor-patient relationship
			Many recommended doctors and nurses should improve their interpersonal skills and take a more holistic, patient-centered approach
			Information provision was problematic for some participants who reported long delays
			Privacy was a significant area of concern for participants, especially females
	Aljughaiman et al. [42]	Adult patients (n = 229) from Dammam	A high level of patient satisfaction was observed
			Patients were more satisfied with the treatment received in public than in private hospitals
			The majority were satisfied with the explanation regarding treatment procedures and prompt answering of their queries in public than private hospitals
	Alghabiwi et al. [43]	Adult patients (n = 253) from Riyadh	The overall level of satisfaction in a doctor-patient relationship was less than moderate
			Patients perceived self-efficacy was significantly associated with the patient’s level of satisfaction with the doctor-patient relationship
			Participants with higher satisfaction with the relationship had a stronger sense of self-efficacy in managing their chronic illnesses
			Trustworthiness was the item that received the lowest score
			Agreement with the doctor on the nature of the medical symptoms received the highest score
	Elagi et al. [44]	General public (n = 830) from Jazan	The majority were aware of the principles and essential role of family medicine, health conditions that family doctors can treat, and conditions they do not treat
			More than half of participants preferred first to seek healthcare from specialists from other specialties
			Although participants were generally satisfied with having a family doctor involved in their care, only a few had a positive experience with family doctors
			Nearly a quarter complained of extended visits and long waiting times in family medicine clinics
	Fothan et al. [45]	Medical Students (n = 210) from Riyadh	Overall, students demonstrated attitudes that favored patient-centeredness
			There were no statistically significant differences identified between students’ demographics and Patient-Practitioner Orientation Scale (PPOS) scores
	Almoallem et al. [46]	Doctors (n = 455) from Riyadh	Disagreement among patients/family and doctors about treatment decision
			Treating patients with impaired or uncertain decision making
			Conflict with administration policies and procedures
			Scarcity of resources in the clinic
			Uncertainty whether to disclose the diagnosis to the patient by delivering bad news
			Conflict on the appropriateness of deciding on a “no-code status” with family or colleagues
			Improperly taken informed consent
			Female doctors were less confident about their knowledge of ethics
	Habbash [47]	Resident doctors (n = 210) from Abha	Older residents have higher scores in communication skills
			The female gender was associated with the best ability to ask questions
			Years of clinical experience were closely related to better communication skills
			Communication skills were affected by the level of residency and attendance at training
	Al-Shehri et al. [48]	Resident doctors (n = 261) from Aseer	There was a gap in knowledge of medical ethics
			The aspects of confidentiality were generally well understood
			Residents responded favorably to questions regarding beneficence and nonmaleficence
			It was confusing to decide whether to always agree to the patient’s wishes or act in the interest of the patient despite their refusal
			Most participants supported informed consent
			Alack of knowledge about abortion ethics and legislation
			Most of the participants agreed to the refusal of examination of a female patient by a male doctor in the absence of a chaperone
			Negative attitude for earning a commission by referring patients for investigations or taking gifts/ incentives from drug companies
	Aljaffary et al. [49]	Patients (n = 345) from Saudi Arabia	Doctors working in public hospitals were more likely to have a higher level of patient trust compared to private hospitals
			Patients with “good” self-assessment health status showed a higher level of trust
			Patients who were treated in private hospitals had a significantly lower score of self-assessment health status than those who were treated in public hospitals
*Informed consent*			
Before delivering medical treatment or carrying out an operative procedure, the legally competent patient’s consent, be he/she male or female, shall be obtained. The doctor shall provide an adequate explanation to the patient or his/her guardian on the nature of the medical treatment or operative procedure he intends to apply (Saudi Ministry of Health,1988)	Al Qarny et al. [33]	Health care workers (n = 224) from Taif	Support for informed consent by health care workers
			Most participants knew that patients should always be told if something was wrong
			Children should never be treated without the permission of their parents or guardians (except in an emergency)
			Almost two-thirds of participants knew that patients not only need to consent for operations but also for tests and medications
	Abolfotouh and Adlan [50]	Adult patients (n = 162) from Riyadh	The overall attitude towards informed consent was positive
			Poor quality of informed consent in terms of experience with the informed consent processes and the transparent delivery of information regarding risks
			More than half of the study sample trusted the doctor to decide on behalf of them
			Many participants were not interested in obtaining a copy of the informed consent
			Quality of scholarly consent score was higher when explained by the doctor and among younger patients
	Darwish and Sabra [51]	Medical interns (n = 87) from Dammam	The majority could recognize the meaning of general and specific consent
			The majority agreed that consent should be given for each new procedure and should be perceived as a continuing process rather than a one-off decision
			The majority decided that surgeons could exceed the consent in case of emergencies and the absence of a guardian
			Less than half of the participants agreed that the patient is allowed to look into his medical record or take a copy of it if asked for that
	Fayez et al. [37]	Health care workers (n = 370) from four tertiary hospitals in Saudi Arabia	The majority agreed that unethical behavior occurred in their workplace
			The confidentiality of patients was compromised
			Informed consent not handled properly
	Hammami et al. [52]	Adult patients (n = 488) from Riyadh	The informed consent process was essential to patients
			Males, pre-procedure, and older patients more favor a self-decision-making purpose
			Females and post-procedure patients more turn an information disclosure purpose
			More self-decision-making and more effective information disclosure was desired
			Mill’s autonomy model of informed consent is preferred, which may be suitable for most patients, especially males and older patients
			Some patients showed a degree of dissatisfaction with the informed consent process
	Almohaimede et al. [53]	Patients (n = 138) from Riyadh	Dissatisfaction among patients regarding the experience of the informed consent process
			Lack of knowledge about the risks of the intervention and alternative management
			Half of the participants wished to be involved in decision-making
			One third had no time to comprehend the information provided
			A higher quality of informed consent was predicted when explained by the doctor
			About half of the participants believed that their decision was not necessary because the doctor had already decided for them
	Alsaihati et al. [54]	Surgeons (n = 140) from Saudi Arabia	Participants had acceptable knowledge about informed surgical consent
			The majority did not give full details to patients about the procedure before taking consent
			There was a careless approach in the consent process among surgeons
			Some considered consent as only a pre-operative routine or just signing the paper
			Some believed that the consent process is strange to Saudi psychology
			The majority were against applying for a license during all surgical procedure
	Mahrous [55]	Patients or family members (n = 176) from Madinah	There is a low level of awareness of patients’ rights
			The consent form followed by complaint registration against a service provided was the best-known patients’ rights variable
			About half of the sample had never heard about patients’ rights
			More males, compared to females, signed a consent form upon hospital admission
	Basharaheel et al. [56]	Surgical doctors (n = 188) from Jeddah	The majority had experience in obtaining informed consent for a surgical procedure
			Senior doctors were the main doctors taking informed consent from patients
			Interns were least exposed to observing surgical procedures than other team members
			Interns were the least comfortable while taking consent
	Alahmad et al. [57]	Nurses (n = 17) from Riyadh and Jeddah	Participants considered parental consent mandatory and necessary from the moment the child is admitted to the hospital and during treatment
			Consent should be easily understandable and have the required information decision
			Obtaining consent from the child’s father reflects a cultural difference between Saudi Arabia and other Middle Eastern countries
	Alsaidan et al. [58]	Patients (n = 246) from Al-Kharj	About 30% of cosmetic procedures were performed without taking informed consent
			Quality of informed consent was generally poor, both in content and administration
			Issues related to lack or poor consent are getting focused only when the procedure ends up with adverse events or non-satisfactions but without actual association
	Alahmad et al. [59]	Doctors, nurses, parents, and medical students (n = 400) from Dammam, Riyadh, and Jeddah	The majority preferred both parents to give consent, followed by either parent without differentiation between parents
			The majority preferred that parental consent forms be detailed enough to obtain the maximum information
			The majority preferred that the form seeking to obtain the permission of the child be short, not to increase the burden on the child since the decision belongs to the parents
			Most participants preferred to rely on a child's level of maturity rather than having reached a certain age so that they could give consent
			A few participants considered the age of 13–14 suitable for a child to give a consent
*Do-not-resuscitate*			
*Fatwa* 12,086: this *fatwa* stipulates that judging resuscitative efforts to be of no avail and issuing a do-not-resuscitate (DNR) order is done by three “specialized and trustworthy” doctors and that the patient’s family or legal guardian is not to be consulted when it comes to issuing the order (General Presidency of Scholarly Research and Ifta in Riyadh, 1998)	Aljohaney and Bawazir [60]	Residents (n = 157) from Jeddah, Makkah, Madinah, and Taif	Most residents participated in DNR discussions with patients and family or surrogate decision-makers
			The most common limitation to meaningful DNR discussions was a lack of understanding of the patient, the patient’s family, or surrogate, followed by inadequate training
			Most residents believed that additional educational programs would enhance their competence in addressing issues related to DNR discussions
			Need for a structured curriculum to teach skills relating to end-of-life issues such as DNR orders to residents in the Saudi medical system
	Amoudi et al. [61]	Interns and residents (n = 140) from Jeddah	A lack of familiarity with DNR policies in local hospitals
			Residents were more familiar with DNR
			Participants failed to affirm whether a clear local or national DNR policy exists
			Participants believed that the patient should be part of the decision-making process
	Al Sheefet al. [62]	Outpatients/caregivers (n = 307) from Riyadh	Half of the participants could define DNR order
			The majority required more in-depth knowledge
			The opinion of the participants regarding the compatibility of DNR order in terms of religion and ethics was divided due to ethical, religious, and medical factors
	Gouda et al. [63]	ER & ICU doctors (n = 112) from Riyadh	Most of the participants were aware of the existence of the DNR policy
			Two-thirds of the participants did not read the detailed policy
			The majority were in favor of having a DNR for themselves in case of a terminal illness
			The majority preferred the DNR order to be a doctor-directed decision
			Every patient should have advance directives
			The most important barriers for initializing and discussing DNR were lack of patient understanding, level of education, and the cultural background of patients
			Most Muslim doctors believe that DNR is not against Islamic rules
	Madadin et al. [64]	ICU doctors (n = 42) from Al Khobar	Participants were aware that DNR in Saudi Arabia is legal
			Cultural standards and religious beliefs do play a role in their decision-making but had less of an effect as compared to other clinical data such as comorbidities, age, and previous ICU admissions
	Baharoon et al. [65]	Patients (n = 300) from Riyadh	A lack of knowledge of the medical condition, advanced planning, and life support
			A will to participate in end-of-life care planning discussions with doctors
			The majority were able to make intelligent judgments about end-of-life decisions
			Limited knowledge of CPR or mechanical ventilation
			90% of participants formed an opinion about the desirability of intensive care
	Kaneetah et al. [66]	Adult patients and the general public (n = 1,693) from Mecca	Most participants wanted to be involved in the decision-making on DNR
			The majority had a lack of knowledge about the DNR practice
			A background in medicine and knowledge about DNR was associated with the acceptance of DNR
			The most common reasons for refusal DNR were of hope and religious concern
	Alsaati et al. [67]	Medical students (n = 429) from Jeddah	The majority believed that there is a *Fatwa* that regulates DNR on the Islamic level
			Most of the participants were familiar with the DNR order
			Lectures in the medical schools on DNR were the primary source of information
			The majority of participants were not sure if there is a clear policy concerning DNR policy at King Abdulaziz University Hospital (KAUH) in Jeddah
			Lack of DNR understanding in patients and their families is one of the most important barriers that impede an effective DNR discussion
			The majority strongly agreed that patients should be involved in DNR decisions
	Al Farhan et al. [68]	Patients (n = 72) from Riyadh	DNR orders were associated with a reduction in doctors providing clinical care
			The drop-in care after DNR was seen only among doctors rather than nurses
			Doctors need more insight into the true goals of DNR orders and should not equate them with withholding other therapeutic interventions
	Al Ahmadi et al. [69]	Participants (n = 400) from Jeddah	Participants with higher educational levels were more familiar with the DNR term
			DNR was poorly understood due to religious and cultural factors
			There were no significant differences among age, gender, and responder status
			The majority of participants chose doctors like the one responsible for the DNR decision
	Aljethaily et al. [70]	Pediatricians (n = 203) from Riyadh	The majority could not define DNR correctly
			DNR policy and procedure were not clear to them
			Half of the participants believed that DNR was a doctor’s decision
			The majority felt that patients had the right to intensive care, despite terminal illness
			A few of samples reported that they would be comfortable discussing DNR with parents
	Almoallem et al. [46]	Doctors (n = 455) from Riyadh	Non-Saudi doctors deciding on life-sustaining treatment or DNR consulted with the ethical committees more frequently than Saudi doctors
			Doctors who received their education and postgraduate training abroad were confident about their ethics knowledge in medical practice but had less confidence in making decisions about life-sustaining treatment or DNR orders
			Consultants compared with non-consultants had more knowledge about ethics, less conflict with family, and were at ease in making decisions about DNR or end-of-life
			Female doctors were less confident about making decisions about life-sustaining treatment or a DNR order
	Abu Yahya et al. [71]	Nurses (n = 157) from Riyadh	Most of the nurses wanted there to be a legal basis for DNR policies
			The majority stated that they wanted to know more about patients’ rights regarding the end-of-life and use of the DNR order
			The majority agreed that DNR orders support the treatment plan for terminally ill patients
*Organ donation & transplantation*			
*Fatwa* No. 99: The Saudi Grand Ulema (1982) addressed the subject of organ transplants, which was unanimously sanctioned. It also sanctioned (by the majority) the donation of organs both by the living and by the dead, who made a will or testament, or by the consent of the relatives (who constitute the Islamic next of kin). The regulations in Saudi Arabia initially restricted genetically related donors or spouses, but many of these restrictions were later removed (Saudi Grand Ulema, 1982)	Alghanim [72]	Residents (n = 897) from Saudi Arabia	Participant from rural areas was less likely to have information about organ donation than their counterparts in urban areas
			More than half of the rural respondents and more than 40% of the respondents living in the urban areas were not willing to donate organs and sign organ donation cards
			The main source of information about organ donation was a TV
			The majority reported that the contribution of health care providers in providing them with knowledge about organ donation and transplantation was “none” or “little”
			Reasons for refusal to donate were worries about receiving inadequate health care after donation, lack of family support, lack of incentives, not enough information about organ donation, fear of complications after organ donation, religion
	Hammami et al. [73]	Adults (n = 698) in the outpatient setting from Riyadh	Most respondents were in favor of posthumous organ donation
			The mandated choice system was the most preferred
			The presumed consent system was the least preferred
			Financial and medical incentives had a negative effect with a predominance of altruistic motives and belief in the sanctity of the body
			No association between favoring a consenting system and age, perceived health status, education level, or knowing an organ donor or recipient
	Mohamed and Guella [74]	Adults (n = 497) from Dhahran	The level of awareness of transplantation and organ donation was high
			The main obstacle related to the concept that having one kidney may only expose the donor to potential medical problems
			Religion was not an obstacle
	Harthi and Alzahrany [75]	Students (n = 400) from Taif	Almost one-third of the participants reported that they had insufficient information about organ donation and transplantation
			No significant association of the willingness to donate with gender or age
			The main source of information regarding organ donation was media, mainly television
			Participants who did not encourage organ donation were concerned about fearing complications and not receiving adequate health care after donation
	Soubhanneyaz et al. [76]	Adults (n = 461) from the Western province	The majority knew well which organs could be donated
			There was a lack of knowledge about the regulations and legislation of organ donation
			The majority were willing to donate organs with no significant differences in males and females
			Religion, money, and age of the recipient appeared to have no role in their will of organ donation
			Participants believed that governmental incentives in the form of monetary and health treatment for donor families and awards would be effective in promoting organ donation
	Almohsen et al. [77]	Students (n = 195) from Qassim	The primary source of knowledge on organ donation was a television
			The majority believed that there is low public awareness regarding the subject
			One-third of students knew about organ donation cards, but none had signed, due to fear of side effects
			There were misconceptions of the Islamic perspective, resulting in fewer donors
			Medical students showed higher knowledge about organ donation cards and the effectiveness of transplantation as treatment compared to non-medical students
	Al Bshabshe et al. [78]	Students (n = 873) from Abha	The majority (92.4%) did not know the religious point of view about brain death and had not heard of any existing decree or *Fatwa* regarding brain death in Saudi Arabia
			Almost half would accept the concept of brain death if one of their relatives had it
			The majority received their information about brain death from the media
			The majority had the impression that there is no difference between brain death and natural death
	Hajjar et al. [79]	Social media users (n = 913) from Saudi Arabia	The majority received information about organ donation from TV and social media
			The contribution of healthcare providers as a source of information was minimal
			An increase in the knowledge of the religious legislation (*Fatwa*) of organ donation
			More than half of the participants were willing to donate their organs
			Reasons for refusal were poor knowledge about organ donation, insufficient support of healthcare providers, religion, lack of family support, and fear of operations
	AlHabeeb et al. [80]	Adults (n = 1298) in 18 cities of Saudi Arabia	Participants accepted the concept of organ donation and were willing to donate
			Concerns remain on heart donation
			Almost a third of participants expressed a fear that healthcare professionals may make less effort to save the lives of potential donors
	Elsafi et al. [81]	Allied health students (n = 434) from Dhahran	Overall knowledge of organ donation was adequate
			The majority were willing to be living donors for their families
			A few participants supported deceased organ donation and thought about an organ donation card
			The most frequent cause of refusal to donate organs was the mistrust of medical staff regarding brain death diagnosis
			Bodily concerns and religion were additional concerns
			Quite a few of respondents supported commercial donation
			The reason to donate organs was to help others and sympathy
			The main source of information was television, relatives/friends, and the Internet
	Sayedalamin et al. [82]	Students (n = 481) from Jeddah	The majority showed a positive attitude towards organ donation
			There were few misconceptions and a lack of knowledge about organ donation, such as organ grafting from a male to a female or vice versa
			The two-thirds were in favor of brain-dead patients donating their organs
			Less than half were ready to donate their organs
			Reasons of refusal to donate were family consent; some wanted to keep all their organs intact, religion and medical history
			The majority knew about organ donation from the media
	Agrawal et al. [83]	Adults (n = 403) from Al-Kharj	Nearly half of the respondents believed that religion does not allow for organ donation
			Less than 3% knew the correct place to go for organ donation
			Participants with more knowledge were more willing to donate their organs
	Al Bshabshe et al. [84]	Students (n = 649) from the Southern province	The knowledge regarding brain death and organ donation was found to be poor
			More than half of the participants have not heard about the term “brain death”
			Less than half of participants were in favor of organ donation from a brain-dead person, ready to donate the organs of a family member or a relative who is brain dead, willing to donate their organs
	Al-Hussain et al. [85]	Adults (n = 409) from Riyadh	More than half of the participants were aware of brain death
			The majority stated that it was acceptable to donate, according to religion
			More than half would agree to donate their organs in case of brain death
			The majority would donate their organs without discussing them with families or friends
	Almufleh et al. [86]	Residents (n = 2,596) from Riyadh	The level of organ donation awareness was high
			Organ donation awareness was found to be more in females, educated individuals, those with higher socioeconomic status, and married participants
			More than half of the sample expressed willingness to donate brain-dead relatives’ organs
			Reasons for refusal to donate were body disfigurement, religion, unawareness, and family disagreement
	AlHejaili et al. [87]	Students (n = 821) from Saudi Arabia	The majority has sufficient knowledge of organ donation and transplantation
			The degree of awareness positively impacted the willingness to donate
			Commonly cited the reason as a barrier to donation after death was the fear of premature termination of medical treatment to facilitate organ retrieval and transplantation
			Females scored higher than males in both the awareness and altruism
	Alsharidah et al. [88]	Adult Saudis (n = 648) from Riyadh	The majority knew the concept and procedure of organ donation
			The majority agreed to sign donation cards by reasons of faith, good deed, importance of donation, and belief that organs are not beneficial after death
			Religion was not a barrier
			There was a lack of awareness about the Saudi Center for Organ Transplantation (SCOT)
	Aziz et al. [89]	Adults (n = 350) from Aseer	Participants showed an acceptable level of awareness about organ donation
			A generally positive attitude towards organ donation for any person regardless of age, religion, mental status, or health status
			Doctors' role as a source of information was poor
			Internet and mass media played the highest role as a source of information
	Alruwaili et al. [90]	Paramedical and medical students (n = 350) from Saudi Arabia	Eye donation awareness and willingness to donate are generally low in Saudi Arabia
			Less than 7% of medical students knew of the existence of eye banks
	Hazzazi et al. [91]	Students (n = 744) from Jazan	A lack the knowledge on hematopoietic stem cell transplantation (HSCT)
			Participants who registered in the Saudi stem cell donor registry (SSCDR) had better knowledge and attitudes towards HSCT than unregistered participants
			Long-term side effects of HSCT were the most common concern of the participants
			The time commitment was the main concern of the registered students
	Alnasyan et al. [92]	Adults (n = 1453) from Saudi Arabia	A positive attitude toward organ donation among the majority
			High rate of willingness to donate correlated to the high rate of educated participants
			The majority believed that consent should be acquired from the donor
			The level of knowledge about SCOT was as low as 12.6%
	Alanazi et al. [93]	Residents (n = 1292) from Saudi Arabia	The majority had no sufficient information about corneal donation and did not know how or where to apply to register as a cornea donor
			A significant correlation between participants’ knowledge and willingness to donate
			The largest perceived barrier was the lack of information on where to donate or register as donors
			The main motive to donate was the religious belief of doing good and being charitable with their organs
	Almutairi [94]	Students (n = 425) from Central province	Medicine and physiotherapy students in their final year scored higher knowledge, attitudes, and willingness towards organ donation compared to dentistry, nursing, and paramedical counterparts
			Females scored higher than males in all the three domains of knowledge, attitude, and willingness toward organ donation
	Alnajjar et al. [30]	Students (n = 113) from Jeddah	The majority were aware of the importance of organ donation
			More than half of the participants expressed a willingness to donate the organs of family members if they were to be diagnosed with brain death
	Alibrahim and Jindan [95]	Adults (n = 1001) from Eastern Province	Participants had poor knowledge about corneal donation
			Less than one-third were favorable to postmortem corneal grafts
			Religion hindered the willingness to donate in about one-quarter of the sample
	Kazzaz and Da’ar [96]	Pediatric intensivists (n = 100) from Central, Eastern, Northern, Western, Southern regions	Low perceived comfort levels in several organ donation competencies
			Comfort levels were influenced by the participants’ frequency of exposure to donation after brain death, the health sector, and region of practice
			Participants viewed most of the competencies as important to their practice
			Low comfort levels with competencies were associated with gaps in knowledge
	Thirunavukkarasu et al. [97]	Students (n = 400) from Jouf	The most common organ that can be donated are kidneys, blood, heart, and eyes
			About two-thirds were not aware of SCOT and its activities
			The government has to promote organ donation to the public
			The majority were willing to donate their organs
			Common barriers for organ donation were a lack of knowledge, founded and unfounded fear, and refusal from family members
	Omran et al. [98]	Students (n = 352) from Jeddah, Mecca, and Taif	Most of the sample had poor knowledge about organ donation
			Higher knowledge in sixth-year students than second-year students
			The majority had an appropriate attitude about organ donation
	Altraif et al. [99]	Adults (n = 376) from Riyadh	A correlation between higher educational level, the knowledge of brain-dead donation, and the Islamic point of view
			More than half have heard about SCOT
			Health-related occupations showed more awareness about organ donation, SCOT, and willingness to donate their families’ organs
			The main barrier for organ donation was lack of information, belief that organ donation disfigures the body and that donated organs can be misused or abused
			Most of the respondents preferred donating to young people
	Gelidan [100]	Adults (n = 698) from Riyadh	Almost all age groups knew about organ donation after death, with male prevalence
			Participants with higher education were more aware of organ donation
			Females were more enthusiastic and had significantly high acceptance of skin donation as compared to males
			Religious factors were the most common reason to refuse skin donation
	Darwish et al. [101]	Adults (n = 1508) from Saudi Arabia	The majority supported organ donation
			More than half were willing to donate any organs
			The majority did not know the organ donation policies
	Al-Oufi and Alghamdi [102]	Adults (n = 670) from Madinah	The majority showed high levels of knowledge about blood donation but poor knowledge about organ donation
			The majority of participants showed negative attitudes towards organ donation
			The main source of knowledge were friends and family
			Knowledge was depended on the education and occupation
	Bukhari [103]	Adults (n = 1099) from Saudi Arabia	Saudi population was willing for organ donation in general and in specific conditions
			Females were more willing to donate their solid organs than males
			The donation was more likely among the young population

### Overall research trends analysis

**Fig. 2:**
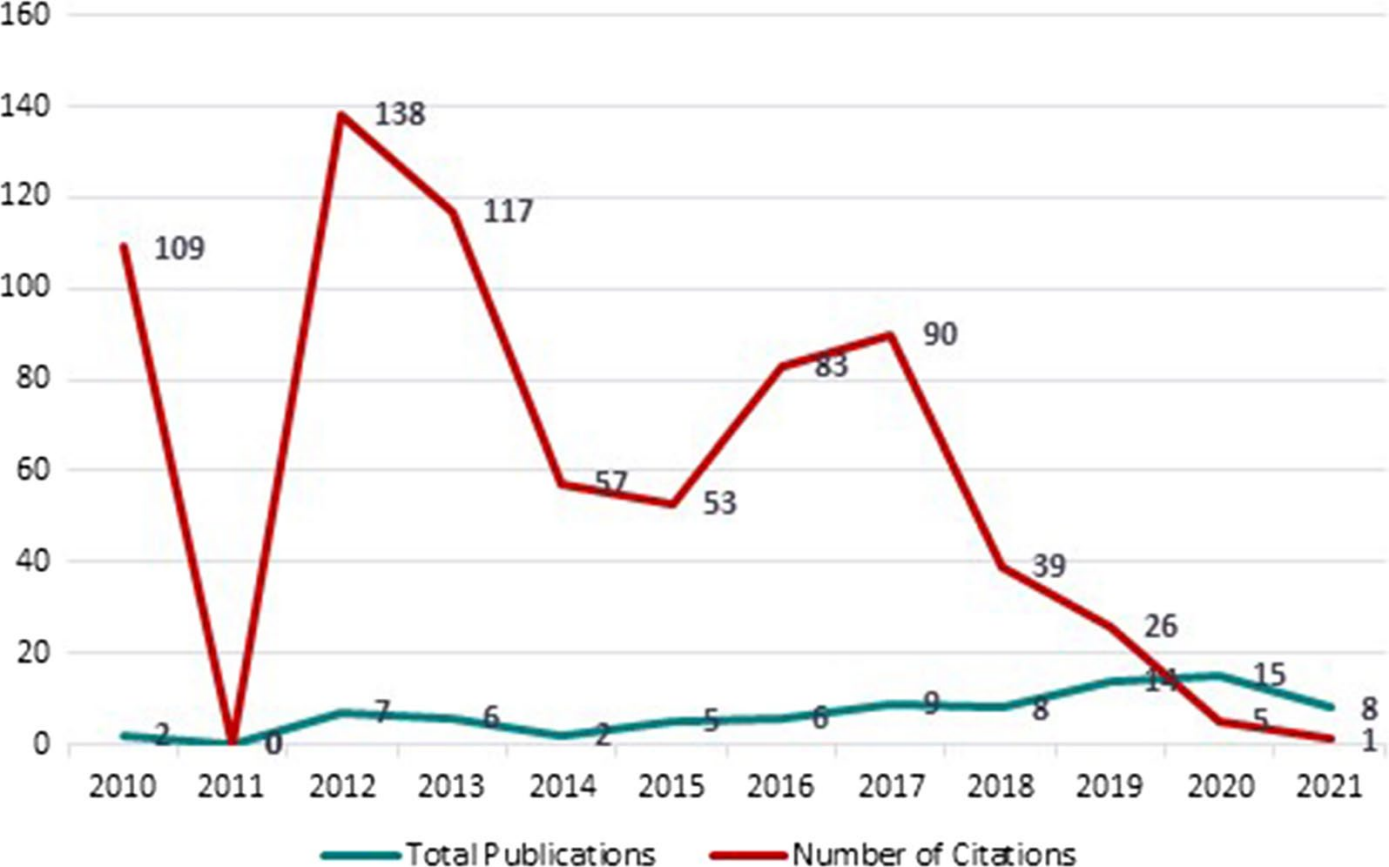
Publishing and citations patterns of medical ethics articles 2010–2021

The total number of publications and citations of the medical ethics literature, based on study objectives, from 2010 to 2021 are shown in Fig. 2. The first five years (2010–2015) have observed little progress, and in 2011 there was no publication as such. However, the highest number of citations falls on articles published from 2010 to 2013. Although since 2017, the growth of publications has been gradually increasing, the number of citations has decreased from 2017 to 2021, with most of the publications having 0 citations. The publications peaked in 2020 (TP = 15), and the number of citations peaked in 2012 (TC = 138).

### Thematic analysis of published articles

**Fig. 3:**
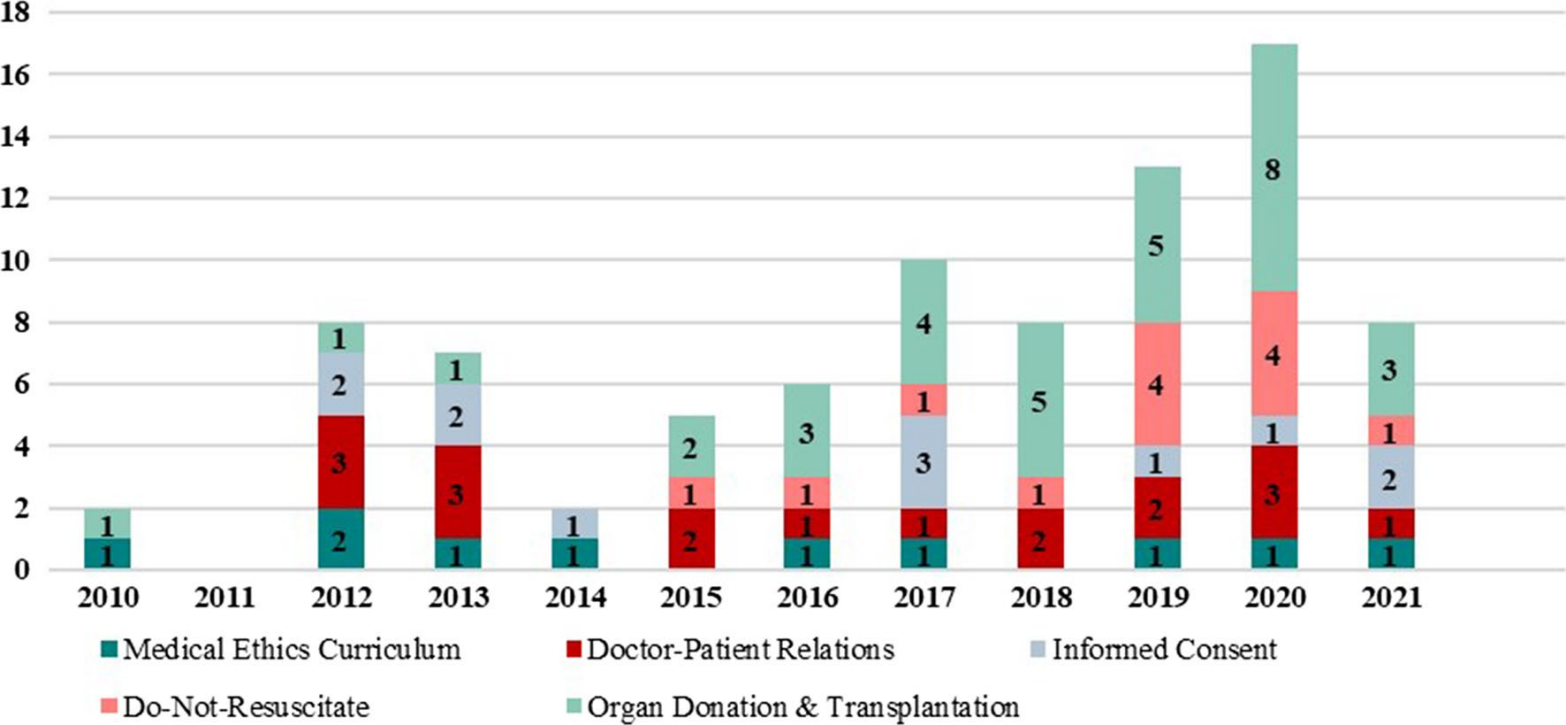
Thematic analysis medical ethics articles 2010–2021

The thematic evaluation of five domains included in this research showed that the most publications were produced focusing on ‘Organ Donation & Transplantation’ with 33 articles, followed by ‘Doctor-Patient Relations’ with 18 publications (Fig. 3). The lowest publications were associated with ‘Medical Ethics Curriculum’ with ten articles. Furthermore, Fig. 3 shows the shift of ‘Do-Not-Resuscitate’ research streams with the highest number of publications released in 2019 and 2020.

### Most productive provinces on medical ethics research

**Fig. 4:**
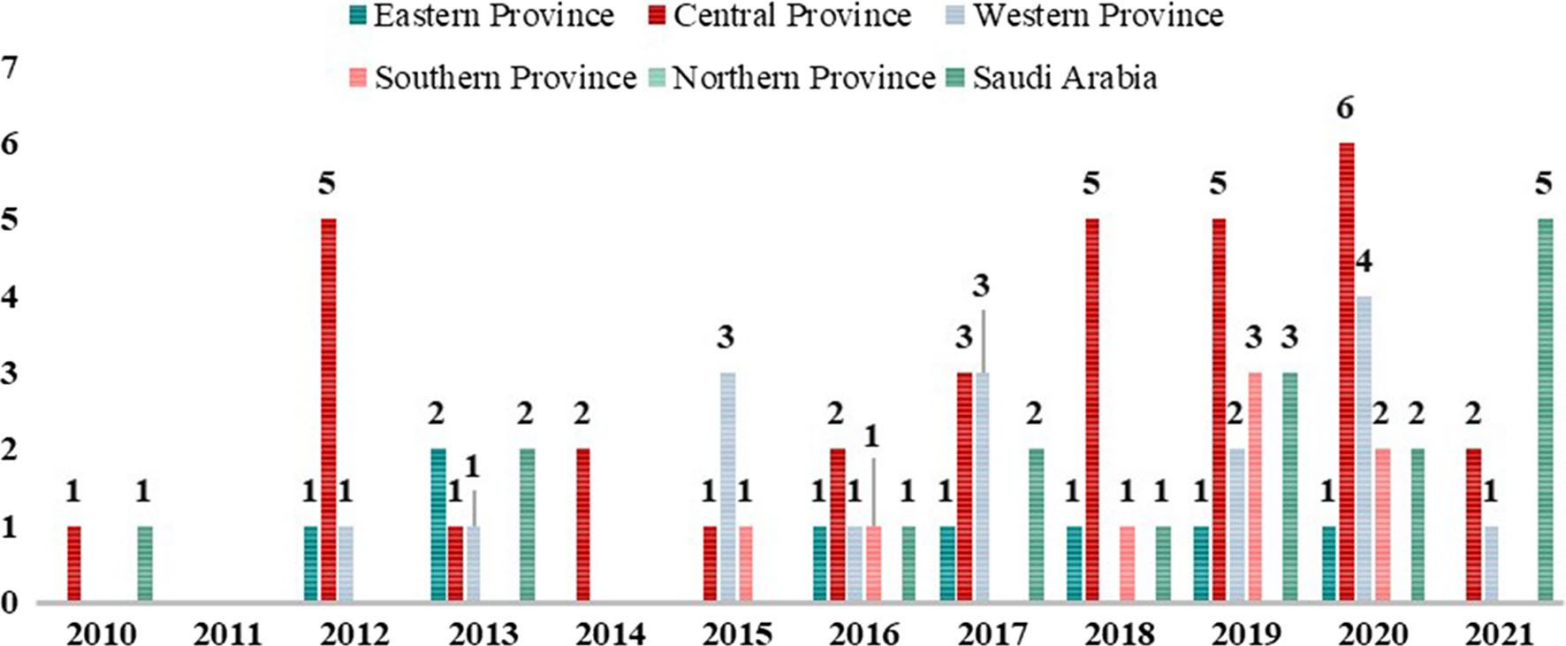
Medical ethics articles publishing patterns for Saudi Arabia and Provinces 2010–2021

Figure 4 presents data on the publication of articles on medical ethics in Saudi Arabia and its provinces. The number of articles targeting Saudis from all provinces of the Kingdom was 17. The Central Province emerged the first in the number of publications (33), followed by the Western province with 16 publications. The Eastern and Southern provinces issued eight articles each, half the number of Western and Central Provinces. Although residents of the Northern province have been included in the articles targeting Saudis from all provinces, no research has been identified where the target population would be residents of the Northern Borders Province only.

### Authorship pattern

**Fig. 5:**
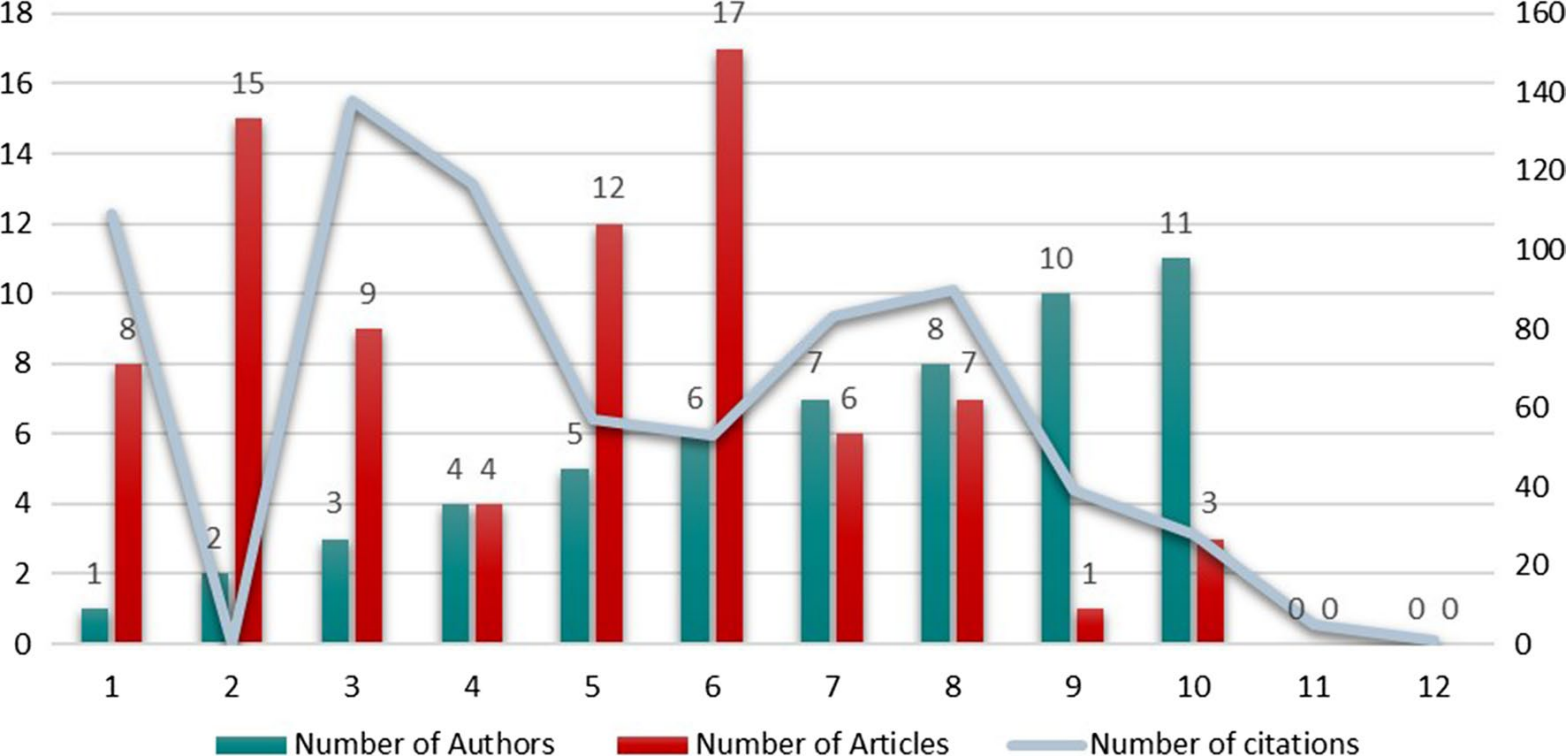
Authorship pattern of medical ethics literature 2010–2021

Figure 5 shows a sample of authorship of medical ethics articles included in this study. The authorship pattern ranges from a minimum of one author to a maximum of 11 authors. Overall, these data indicate that medical ethics research has followed a collaborative approach in most cases involving two authors (15) and six authors (17). In addition, there are four publications with ten or more authors.

### Highly cited articles on medical ethics research

**Fig. 6:**
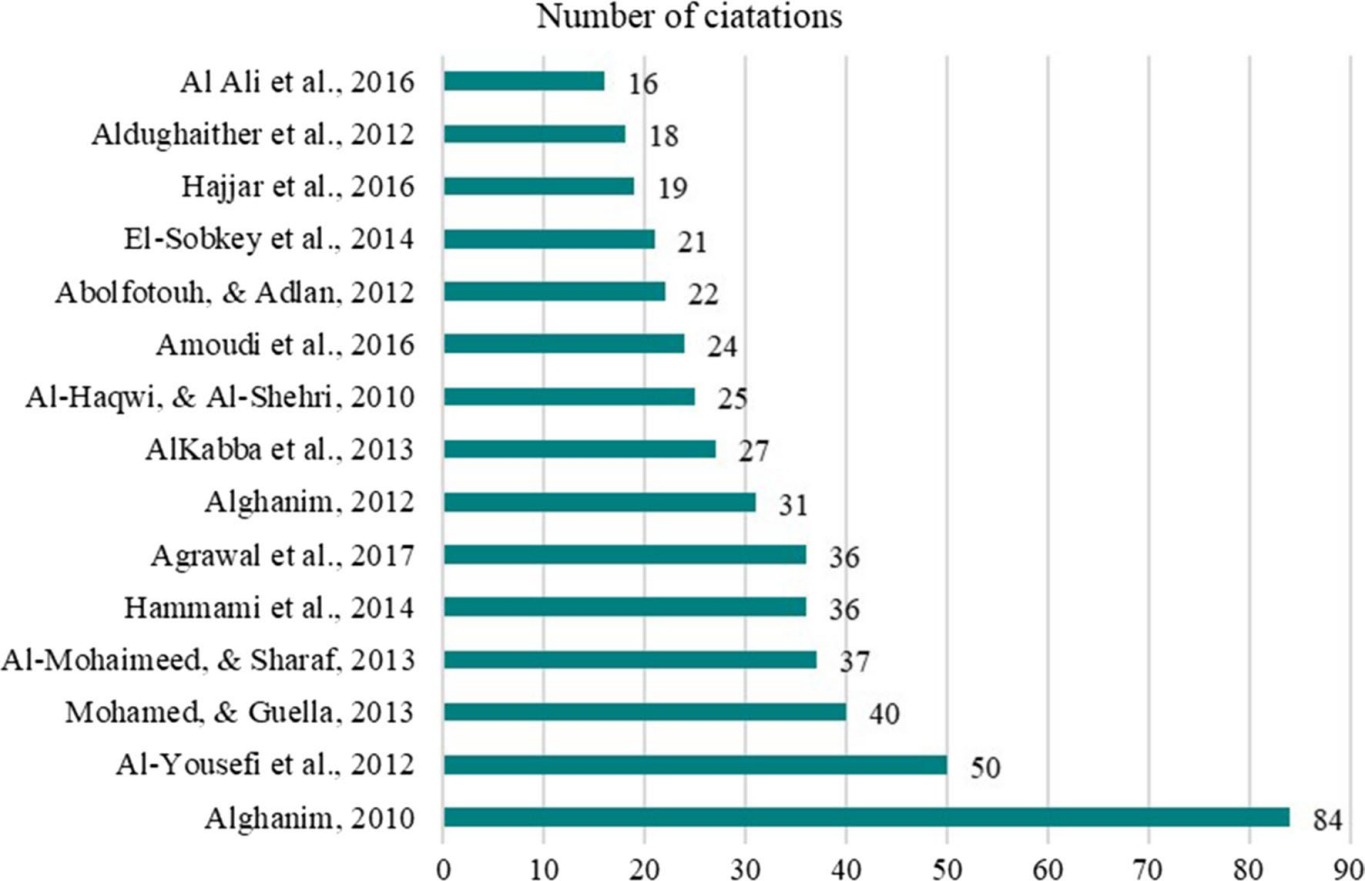
Top 15 most cited articles on medical ethics in Saudi Arabia 2016–2021

Figure 6 presents the bibliographic information of the top 15 highly cited medical ethics articles included in this research. The top 15 highly cited articles’ citations and years ranged a maximum of 16 to 84 citations from 2010 to 2016. One article obtained 84 citations by Alghanim, S.A., 2010, entitled “Knowledge and attitudes toward organ donation: a community-based study is comparing rural and urban populations.” This article was followed by an article written by Al-Yousefi, N.A., 2012, entitled “Observations of Muslim doctors regarding the influence of religion on health and their clinical approach.” The article obtained 50 citations. A thematic evaluation of the five domains included in this study found four articles in each of the following areas were among the most cited: ‘Organ Donation & Transplantation,’ ‘Doctor-Patient Relations,’ and ‘Medical Ethics Curriculum.’ However, despite publication growth in ‘Do-Not-Resuscitate’ research, only one article in this area has appeared on the most-cited list. This article was cited 24 times and was written by Amoudi, A.S., Albar, M.H., Bokhari, A.M., Yahya, S.H. and Merdad, A.A., 2016, entitled "Perspectives of interns and residents toward do-not-resuscitate policies in Saudi Arabia." Remarkably, 24 of the 82 included articles received zero citations, with 11 published in 2020 and 7 in 2021.

### Keyword analysis of medical ethics research in Saudi Arabia

**Fig. 7:**
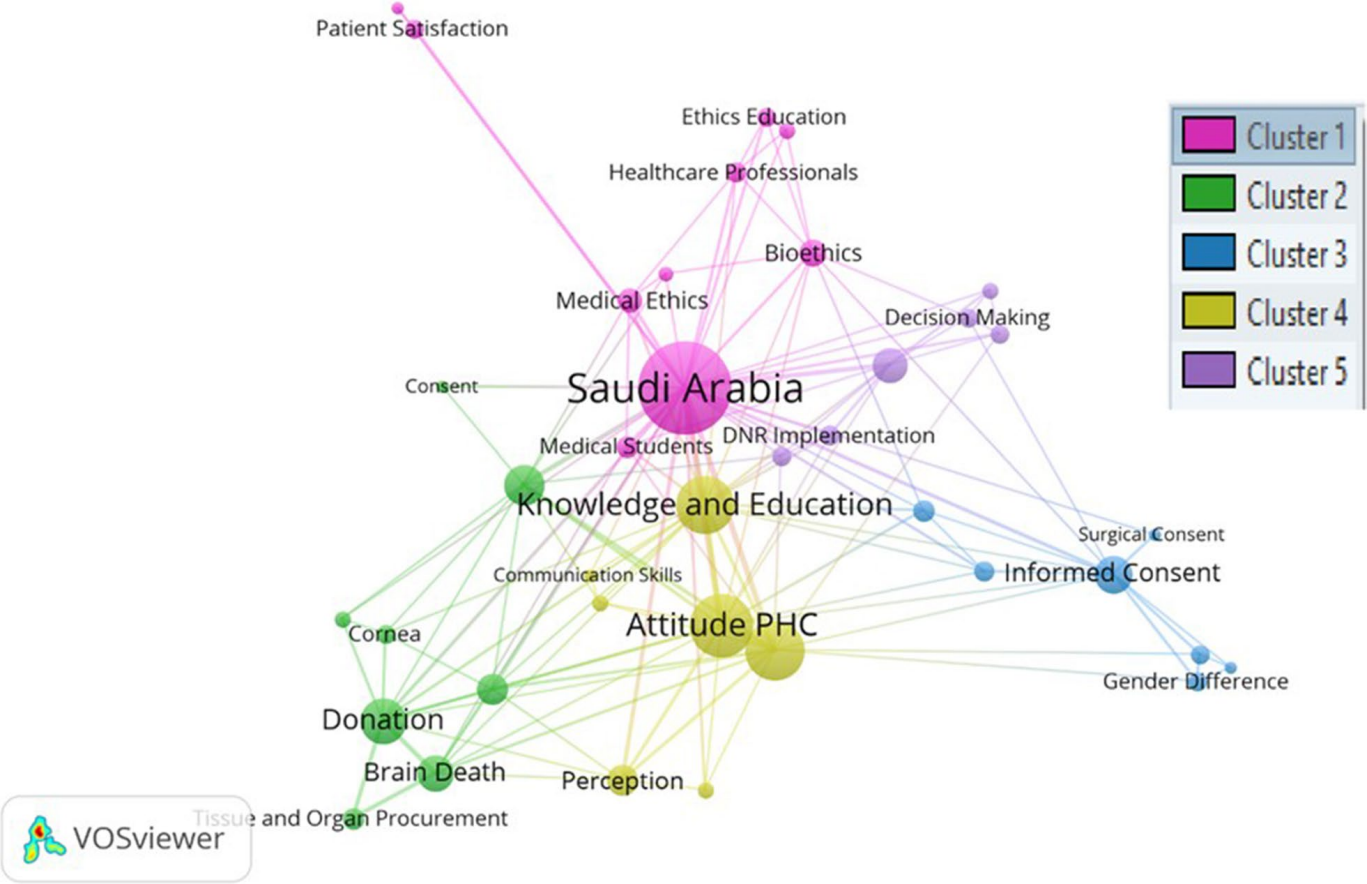
Co-occurrence network of keywords used by the authors (2010–2021)

The next cluster of keywords (Fig. 7) demonstrates that the issue was constantly discussed in the studied articles under consideration between 2010 and 2021. Each color represents a separate cluster, and clusters are arranged based on link strength and occurrence. Co-occurrence network of author keywords was a minimum number of occurrences: three (i.e., consent, DNR implementation, medical ethics, patient’s rights).

There are five clusters (indicating various colors) having a relationship. Cluster one is the strongest network relationship, followed by 2–5 clusters, respectively. Hence, the size of the bubble indicates the nature of the relationship with link strength and occurrence. The five keywords with the highest total link strength are ‘Saudi Arabia’ (link strength: 61), ‘attitude PHC’ (39), ‘organ donation’ (32), ‘knowledge and education’ (30), and ‘donation’ (21). The results confirm the importance of knowledge and education among health care providers and the general public to improve the attitudes towards medical ethics issues and concerns.

## Discussion

This systematic review is the first organized synthesis of the five most debated bioethical domains that have been studied in the Kingdom of Saudi Arabia. Eighty-two studies were identified across five main domains for a final review and assessment: 'Medical Ethics Curriculum in Saudi Arabia,' 'Doctor-Patient Relations,' 'Informed Consent,' 'Do-Not-Resuscitate,' 'Organ Donation & Transplantation.' To the authors' knowledge, there are no similar systematic reviews in bioethical research in the Kingdom of Saudi Arabia, and therefore the direct comparison is not possible.

### Medical ethics curriculum

In the past two decades, the discussion of bioethical issues in Saudi society has become increasingly important. However, it would be fair to note that there is still limited guidance on teaching and learning bioethics, which will inevitably have a tangible impact on society in the future [1, 2]. Despite the researchers’ growing interest in medical ethics, this systematic review has shown that from 2010 to 2021, only ten articles were published studying the ‘Medical Ethics Curriculum’ in KSA [22–31]. Of these ten articles, the research by El-Sobkey et al. (2014) is in the list of the most cited articles with 21 citations.

Overall synthesis of this domain showed that regardless of the region, target population, most participants in all included studies agreed on the importance of studying the principles of medical ethics as a discipline with its methods, literature, vocabulary, and content [22–31]. Although some participants completed theoretical ethics classes while attending medical school, the theory does not address the practical ethical dilemma faced in daily practice after graduation [25, 27–29, 31].

Thus, there was a lack of knowledge about organ donation regulations, withholding or stopping mechanical ventilation, conflict with family, and advice from the ethics committee, religious aspects, brain death, DNR policies, the existence of the Saudi PBR [23, 24, 26, 27].

The conclusions and recommendations of all included articles were consistent, stating that teaching bioethics is a complex and long-term process that helps graduate and undergraduate students embrace the roots of their culture, knowledge, and principles [22–31]. While there is no single best model for teaching medical ethics, teaching bioethics is essential to educating medical students by developing a comprehensive bioethics curriculum. Students are encouraged to participate in all processes actively [22–31]. The learning outcomes for each activity should be used as a guide for assessing the adequacy of the bioethics curriculum, together with an assessment of effectiveness.

### Doctor-patient relations

The thematic evaluation found that 'Doctor-Patient Relations' with 18 publications was the second most studied domain. The synthesis of the included studies on medical ethics knowledge showed that most participants, mainly healthcare representatives had little knowledge about doctor-patient relations [32, 33, 36, 41, 42, 44, 45, 48]. However, there was a strong consensus that every patient should be treated with honesty and dignity. Patients of a high socioeconomic class should not be treated with extra care, and confidentiality should be maintained in all circumstances [32, 33, 36, 41, 42, 44, 45, 48].

Lack of training, knowledge of cultural norms, patient participation in decision-making, gender differences between patients and doctors, and a lack of time were major barriers to effective communication skills with patients and their families [32, 34, 37–40, 43, 46]. Patients recommended doctors and nurses to improve their interpersonal skills and take a more holistic, patient-centered approach. This can improve information delivery and resolve disagreements between patients/family members and healthcare providers about treatment decisions [32, 34, 37–40, 43, 46].

The doctor’s specialty influenced communication patterns and patient satisfaction. Family doctors were more closely associated with building rapport, psychosocial exchange, and patient orientation than other specialties. Senior medical specialists were more confident in their communication skills [34, 35, 38–41, 46–49]. Doctors working in public hospitals were more likely to have higher patient trust than private hospitals. Despite the general synthesis of results confirming patient satisfaction with explaining treatment procedures and the prompt response to their questions, some patients perceived the nurses as insufficiently responsive and compassionate [34, 35, 38–41, 46–49].

As shown, the doctor-patient relationship is a complex area made up of several factors, including doctor-patient communication, patient participation in decision-making, and patient satisfaction. Hence, in most cases, treatment is based on this relationship [2, 4, 7, 8]. The doctor and patient are expected and recommended to work together to improve psychopathological conditions, with particular attention to therapeutic relationships that affect the patient's thoughts, emotions, and behavior [32–49].

### Informed consent

The third most studied domain of medical ethics research in Saudi Arabia was 'Informed Consent’ with 12 publications. Health care providers supported informed consent. The majority agreed that consent should be given for each new procedure and should be perceived as an ongoing process, not a one-off decision, with parental consent being considered mandatory for children's treatment. However, there was low quality of informed consent in terms of experience with informed consent processes and transparency of risks [33, 37, 50, 51, 55–57, 59].

Although patients’ opinions on the purpose of informed consent varied, the informed consent process and being aware of treatment was important for all [50, 52, 53, 55–57, 59]. In terms of the trust, some patients trusted the doctor to make decisions on their behalf, while others required more independent decision-making and preferably more effective disclosure of information. In addition, there was an overall poor quality of informed consent process and administration, knowledge of the risks of intervention and alternative treatment, and insufficient information included in informed consent [50, 52, 53, 58].

Remarkably, a study by Alsaihati et al. (2017) among n = 140 surgeons in Saudi Arabia found that while there was sufficient knowledge about informed consent to surgery and how to obtain it, most surgeons did not fully inform patients about the procedure before obtaining consent. Some saw consent as just a preoperative routine or simply signing a document because the consent process is strange to Saudi psychology. The majority were against the use of consent for all surgical procedures [54].

Informed consent is a standard procedure for human studies involving individually identifiable data. The principle of informed consent is based on patient autonomy, which is explained as the legal embodiment of the idea that everyone has the right to make decisions that affect their well-being [6, 37, 52]. The Saudi Council for Health Specialties has developed guidelines on informed consent as part of the ethics of the medical profession. However, doctors' and patients' levels of awareness and adherence to these guidelines in Saudi Arabia remain unclear and controversial. Thus, these findings create a basis for introducing formal informed consent training to make written information more accessible to doctors and patients [6, 54].

### Do-not-resuscitate

The synthesis of results showed that most healthcare representatives could define the order of DNR. Still, it required deeper knowledge to learn whether there is a clear local or national DNR policy. Those who knew about the policy did not read in detail [60–63, 67, 68, 70]. The most common limitation of essential discussions about DNR was a lack of patient understanding, educational level, cultural background of patients, and a lack of DNR policy knowledge by medical staff [60–64]. Most health care representatives wanted to learn more about patients' rights regarding end-of-life care and the use of the DNR order, as this would support the treatment plan for terminally ill patients [60, 62, 67, 68, 71].

Studies in which patients were included as a target population reported a lack of knowledge of the medical conditions for DNR practice [62, 65, 66, 68, 69]. Patients expressed a will to participate in discussions with doctors on planning end-of-life care and making their own decisions. Patients with higher levels of education, medical background, and knowledge of DNR were more likely to agree with DNR practice [62, 65, 66, 68, 69].

Non-Saudi doctors making decisions about life-sustaining treatment or DNR orders consulted with ethics committees in their hospitals more often than Saudi doctors [46]. Saudi doctors who received their education and postgraduate studies abroad were confident in their knowledge of ethics in medical practice but were less confident in making decisions about life-sustaining treatment or DNR orders [46].

Saudi Arabia's DNR Policy is a binding legal document formulated following the provisions of Islamic law, with a focus on Saudi patients, cultural background, and social needs [6, 10]. However, it has been shown that more efforts are required to improve and optimize end-of-life care and DNR policies by educating and training healthcare personnel and the general public.

### Organ donation and transplantation

The most studied domain of five included domains was ‘Organ Donation and Transplantation,’ 33 articles, with the most papers being published in 2020. Alghanim (2010) wrote the most cited article on knowledge and attitudes toward organ donation [72].

The synthesis of organ donation and transplantation data has led to several general conclusions reported by different authors. Thus, the overall knowledge about organ donation and transplantation varied based on the research objective (i.e., blood, skin, kidney donation). In general, the participants reported insufficient information about organ donation and transplantation [72, 75, 78, 90, 91, 95, 96, 102]. The main source of information about organ donation was TV, social media [72, 75, 77, 79, 81–84, 89].

Participants from rural areas were less likely to have information about organ donation than their counterparts in urban areas [72]. Organ donation awareness was higher in educated individuals with higher socioeconomic status and married participants [86, 87, 94, 99, 100, 102, 103]. The degree of awareness was found to impact the willingness to donate positively. Reasons for refusal to donate were the fear of premature termination of medical treatment to facilitate organ retrieval and transplantation, worries about receiving inadequate health care after donation, lack of family support, lack of incentives, not enough information about organ donation, fear of complications after organ donation, religion [72, 75, 79–83, 86, 95, 97, 99, 100].

In contrast to the stated barriers to organ donation, among those participants who expressed their will or were positive about organ donation and transplantation, gender, age of the recipient, religion and incentives did not appear to play a role. Moreover, organ donation was motivated by helping others and compassion, a good deed, the importance of donation, belief that organs are not beneficial after death, and an altruistic act [30, 73–76, 81, 85, 87–89, 93, 98, 101, 103].

Despite the Saudi Center for Organ Transplantation (SCOT) activities since 1985, there was a lack of knowledge on where to go for organ donation [83, 92, 97]. Thus, in the study by Alnasyan et al. [92], the level of knowledge about SCOT was only 12.6%. This is consistent with Thirunavukkarasu et al. [97], where about two-thirds of the study participants were unaware of SCOT and its organ donation activities. Furthermore, in an earlier study by Agrawal et al. (2017), less than 3% knew the correct place to go for organ donation [83].

Organ transplantation is a life-prolonging and life-saving medical procedure in which an entire or partial organ of a deceased or living person is transplanted to another person. Islamic guidelines and Islamic law describe certain rules and regulations that allow organ transplants to be performed [6, 11, 12]. Organ donation is an act of mercy, benevolence, altruism, and love for humanity. Nonetheless, as can be seen from the literature cited, organ donation remains a very personal yet complex decision involving medical, legal, religious, cultural, and ethical concerns [6].

This bibliometric analysis was limited to five domains of bioethics within Saudi society. However, a literature search identified a number of articles addressing ethical issues related to the COVID-19 outbreak, such as the distribution of intensive care unit (IC) beds, digital tracking applications, vaccine distribution, the ethics of the general risks of COVID-19, risks in vaccine supply chain infrastructures, ethical issues in patient care, among others [104–107]. Hence, this study could start a series of systematic reviews that will explore all areas of bioethics in the Saudi health care system. Particular attention should be paid to study bioethical issues during COVID-19 pandemic [104–107].

## Strengths and limitations

The strengths of the study design include its systematic approach, an explicit, transparent, and reproducible approach that has been adapted, recommended, and used by previous researchers. However, research design comes with potential limitations. Although researchers thoroughly searched databases to include all relevant articles, there is a chance that several important studies may have been overlooked. Thus, most literature on included domains have been written in Arabic, whil this study was limited to English literature, causing a significant deficiency in including more studies, which ultimately affected the results and conclusion.

As mentioned, many *fatwas* govern bioethical research in different domains. However, including all domains in one systematic review would be incomprehensible and a source of bias. Therefore, based on this study, a new systematic review could be initiated to address areas such as stem cell research, genetic and biobanking ethics, cloning, and infertility treatment, among others.

This study of medical ethics in Saudi Arabia offers new avenues for future research even with limitations. Researchers and policymakers can formulate strategies based on these data. Furthermore, authors identified evolving themes that have received little attention in previous research, including assisted reproductive technologies; medical, surgical, and ethical dilemmas of Siamese twins; hermaphroditism; genetic diseases. It is recommended that future research directions based on this bibliometric analysis of literature include comparative studies from developed and developing countries using more comprehensive demographic variables.

## Conclusion

The overall aim of this study was to systematically identify, compile, describe and discuss ethical arguments and concepts in the five most-studied domains of bioethics in Saudi Arabia and present cultural, social, educational, and humane perspectives. From 2010 to 2021, 82 articles were found to be eligible. Most of the publications with the highest citations were from the Central Province. The articles were mainly published in the ‘Organ Donation and Transplantation’ domain. This systematic quantitative synthesis is expected to guide researchers, funders, and policymakers about the strengths and gaps in knowledge and attitudes regarding medical ethics in Saudi Arabia, both among the general public and health professionals.

## Data Availability

All data generated or analyzed during this study are included in this published article.

## Abbreviations

DNR: Do-not-resuscitate
SCOT: Saudi center of organ transplantation
PSMCHS: Prince sultan military college of health sciences
PHC: Primary health care

## Footnotes

1 Fatwa—religious decisions that are followed by healthcare providers in the Muslim world.^2.^

2 Ifta'a—A council charged with issuing religious verdicts fatwas, on matters that require deep study and interpretation of religious texts.^2.^
